# Boosting Slow Oscillations during Sleep to Improve Memory Function in Elderly People: A Review of the Literature

**DOI:** 10.3390/brainsci10050300

**Published:** 2020-05-15

**Authors:** Federico Salfi, Aurora D’Atri, Daniela Tempesta, Luigi De Gennaro, Michele Ferrara

**Affiliations:** 1 Department of Biotechnological and Applied Clinical Sciences, University of L’Aquila, 67100 L’Aquila, Italy; federico.salfi@graduate.univaq.it (F.S.); daniela.tempesta@univaq.it (D.T.); 2 Department of Psychology, Sapienza University of Rome, 00185 Rome, Italy; aurora.datri@uniroma1.it (A.D.); luigi.degennaro@uniroma1.it (L.D.G.)

**Keywords:** slow oscillations, memory consolidation, acoustic stimulation, so-tDCS, aging, sleeping brain stimulation

## Abstract

Sleep represents a crucial time window for the consolidation of memory traces. In this view, some brain rhythms play a pivotal role, first of all the sleep slow waves. In particular, the neocortical slow oscillations (SOs), in coordination with the hippocampal ripples and the thalamocortical spindles, support the long-term storage of the declarative memories. The aging brain is characterized by a disruption of this complex system with outcomes on the related cognitive functions. In recent years, the advancement of the comprehension of the sleep-dependent memory consolidation mechanisms has encouraged the development of techniques of SO enhancement during sleep to induce cognitive benefits. In this review, we focused on the studies reporting on the application of acoustic or electric stimulation procedures in order to improve sleep-dependent memory consolidation in older subjects. Although the current literature is limited and presents inconsistencies, there is promising evidence supporting the perspective to non-invasively manipulate the sleeping brain electrophysiology to improve cognition in the elderly, also shedding light on the mechanisms underlying the sleep-memory relations during healthy and pathological aging.

## 1. Introduction

In recent years, the knowledge of the physiological mechanisms underlying sleep-dependent memory consolidation has substantially progressed. Considering the functional relationship between slow wave activity (SWA) and post-sleep mnemonic performance, the perspective to promote and enhance the slowest component of slow waves (i.e., slow oscillations; SOs) during sleep to improve cognitive functions is becoming a promising research field. Accordingly, the literature on this topic is rapidly growing (for recent reviews, see [1,2,3]). Among the several contexts in which the SO enhancement can be applied, one of the most intriguing is the elderly population, which is typically marked by a decline of sleep quantity (mostly, concerning slow wave sleep) and quality, as well as of the memory functions. Furthermore, studying the manipulation techniques of the sleeping brain may allow us a better understanding of the actual relationship between sleep-dependent memory consolidation processes and healthy and pathological aging.

In this review, after briefly addressing the state of the art of the current knowledge about the relationship between sleep and memory consolidation and its evolution during aging, we focused on those stimulation procedures finalized to boost SOs and memory consolidation during sleep in the elderly population. In particular, two distinct stimulation approaches are attracting special attention of the scientific community: acoustic stimulation and slow oscillatory-transcranial direct current stimulation (so-tDCS). We described in detail the current literature on this topic, highlighting the potentiality as well as the limitations of the stimulation protocols, finally providing some considerations about the future directions of these fascinating stimulation approaches during sleep.

## 2. Sleep and Memory Consolidation

The current literature is unanimous in conferring on sleep a crucial role in the process of memory consolidation. More specifically, the ability to strengthen and stabilize traces of episodic and declarative memories has been related to the deepest stage of sleep, namely slow wave sleep (SWS) [4,5]. A model proposed to explain the mechanism underlying sleep-dependent memory consolidation is the “active system consolidation hypothesis” (ASCH) [6,7]. Central to the ASCH is the hippocampo–neocortical dialogue. During SWS, memory representations are repeatedly and spontaneously reactivated within hippocampal networks, resulting in sharp wave-ripple activity. The depolarizing ‘‘up-states’’ of the slow oscillations (<1 Hz) are proposed to drive the synchronization between hippocampal ripples (80–130 Hz) and thalamocortical spindles (12–16 Hz) [4,8]. The precise temporal coordination of these non-rapid eye movement (NREM) sleep oscillations is thought to support long-term memory storage, resulting in a gradual relocation of memory traces from the hippocampus to the neocortex [4,6,7]. An alternative model proposed to account for the memory benefit of sleep is the “synaptic homeostasis hypothesis” (SHY) [9]. The SHY claims that the slow wave activity during sleep would be the manifestation of a global synaptic downscaling process aimed at the desaturation of synapses, to allow new subsequent learning. In this view, the specific strongly activated memory traces during wakefulness would be enhanced indirectly, being spared from the process of global synaptic depression. 

However, the two theories are not mutually exclusive and could be unified in a general model [1,7]. The well-known memory benefit of sleep could rely complementarily on both direct (specific synaptic strengthening) and indirect processes (general synaptic downscaling). Within this theoretical framework, the pivotal role of SOs in the relationship between sleep and memory consolidation prominently emerges, being a crucial feature of both the models.

## 3. Sleep-Dependent Memory Consolidation in Older Adults

With advancing aging, sleep undergoes a wide range of substantial quantitative and qualitative changes [10]. Typically, aging is characterized by a decline in total sleep time and sleep efficiency [11,12], longer sleep onset latency, increased wake after sleep onset and sleep fragmentation [13,14,15]. On the other hand, daytime naps become more frequent, potentially impacting the subsequent nocturnal sleep [16]. Another peculiar consequence of aging on sleep architecture is a dramatic reduction of the visually scored SWS [11], especially within the early part of the night. In particular, this decline is accompanied by a reduction in electroencephalogram (EEG) slow waves amplitude [17,18]. Furthermore, a decrease in density and amplitude of sleep spindles has been documented [19,20,21]. According to the crucial role of SWS in the consolidation of hippocampal-dependent memories, the expectation is that the dramatic SWS decline in older adults is associated with the age-related impairment of declarative memory consolidation [22]. Nevertheless, recent literature has provided negative evidence in support of such assumption [23,24], inspiring the hypothesis that aging may go toward a weakening of the functional relationship between sleep physiology and memory consolidation [15,23,25]. 

On the other hand, considering the amplitude decrease of slow waves during aging, SWS may prove to be an unreliable index to account for the relationship between sleep and memory consolidation processes. This consideration led to the idea of focusing on EEG power instead of the traditional sleep stage measures for studies on elderly populations [26]. Actually, SWA (0.5–4 Hz) shows a quite robust association with declarative memory performance in older people [27,28]. Aging is marked by medial prefrontal cortex (mPFC) atrophy, which is a crucial generator of slow waves during sleep [29]. Mander and colleagues [28] proposed that mPFC atrophy could underlie the age-related memory consolidation deficit through the disruption of SWA [30]. Furthermore, the mPFC atrophy has been linked to the functional uncoupling of slow waves and spindle activity, which has substantial consequences on the mechanism of hippocampal-dependent memory consolidation [31].

## 4. Current Approaches to Improve Memory Function in Elderly Population Enhancing Slow Oscillations during Sleep

### 4.1. Acoustic Stimulation

The presentation of auditory stimuli during NREM sleep can lead to a firing phase synchronization of wide neuronal populations, which results in an increase in SOs and SWA [32]. In this field, most studies adopted pink noise as acoustic stimulus, considering the particular capacity of this sound to promote the synchronization of brain waves during sleep [33]. In particular, studies with this approach have used low-frequency rhythmic stimulation (<4 Hz) aimed to promote SOs [34,35]. Although rhythmic stimulation seems to be effective to drive EEG modifications, the current literature has provided insight about the importance of the timing of such stimulation to optimize the effects on the electrophysiological level and to extend the benefits to memory domain [32,36]. Accordingly, the up-state of the SOs has proven to be an ideal candidate for this purpose, considering the implication of the SO up-state in the sleep-dependent memory consolidation [37]. In fact, the depolarized up-state is proposed to orchestrate all that set of brain rhythms during sleep implied in hippocampus-dependent memory consolidation, such as hippocampal ripples and thalamocortical spindles. On the other hand, studies addressing the consequences of acoustic stimulation during the down-state of SO showed a disruption effect on the subsequent SOs, failing to report any memory benefit [37,38]. This knowledge led to the development of advanced systems based on algorithms to track online specific phases of the EEG signal, allowing to automatically deliver brief acoustic stimuli specifically during the up-state of SOs, or during the transition from the down- to the up-state of the SOs [37,39,40]. This approach, termed closed-loop auditory stimulation, has proven to be effective in increasing SWA power, augmenting both amplitude and quantity of SOs, and boosting spindle activity phase-locked to the SO up-state. Remarkably, the cognitive benefits of this procedure have been reported in young adults [1,2]. 

Considering this growing literature, the next step consisted of testing the potential feasibility and efficacy of this approach in older adults (Table 1), typically characterized by a decline both in slow waves amplitude and memory function. For this purpose, Papalambros and colleagues [41] were the first to test the effect of one night of acoustic stimulation on both electrophysiological and behavioral measures on thirteen cognitively healthy older subjects (mean age, 75.2 years; range 60–84). Adopting a randomized crossover design, the authors compared the consequences of one entire night of acoustic stimulation versus one night of sham stimulation on declarative memory performance. The memory domain was assessed through a word-paired association task (WPAT), widely used in the literature to measure hippocampal-dependent mnemonic processing (e.g., [26,37,42]). In order to evaluate the effect of the two experimental conditions, morning performance was compared to evening scores. During the stimulation night, sounds were delivered during the transition from down- to up-state of the SO (20 degrees before the peak of the up-state) by using an automated phase-locked loop system (PLL) [40]. Of note, although the primary target of this detection system is the SOs, it has flexibility within the entire delta range (0.5–4 Hz). According to the authors, this feature allows optimizing the stimulation complying with the variability of the slow wave frequency in the elderly. Pulses of pink noise (50 ms) were delivered through headphones and their intensity was subjectively adjusted so that sounds were not evaluated as disturbing (30–50 dB). Blocks of five sounds separated by an average of 1.2 seconds were administered, alternated with an equivalent time period (6 seconds) of pause. In line with the hypothesis, participants showed a significant memory improvement after the stimulation condition compared to the sham condition, replicating the results obtained in young adults [37]. Sleep stage scoring did not highlight any significant impact of stimulation on sleep architecture. Additionally, perceived sleep quality did not differ between the two experimental conditions. As far as the electrophysiological effects are concerned, the stimulation induced an enhancement of spindle density and amplitude. Moreover, sound presentation led to an amplitude increase of SOs, and an average increment of SWA in the 6-second blocks of stimulations compared to periods of pauses during the stimulation night. This within-block enhancement of SWA was positively correlated to memory improvement. Nevertheless, there were no differences between the two experimental conditions on average SWA of each NREM period, nor any association between NREM SWA and memory performance. These results led the authors to interpret the stimulation-induced memory benefit from the perspective of a qualitative transient reorganization of SWA, rather than of a general SWS or SWA enhancement. This reorganization would emerge as a crucial component able to trigger neural synchronization within the thalamocortical network, which supports sleep-dependent memory consolidation. 

In light of these encouraging results, the same research team went further, applying the same procedure to an elderly group affected by amnestic mild cognitive impairment (aMCI) [43]. This condition is characterized by mnemonic deficit, and it is conceived as a probable precursor of dementia and Alzheimer’s disease [44]. This clinical population is marked by various sleep irregularities, such as a further decline of SWS [45,46], SWA [27] and spindle density [47]. Using the same randomized crossover design and stimulation procedure of Papalambros and colleagues [41], nine aMCI participants (mean age, 72 years; range 62–86) were exposed to one night of PLL acoustic stimulation, compared to one night of sham stimulation. Again, memory performance was assessed with WPAT, complemented by a test battery [48] encompassing a multidimensional cognitive evaluation (working and picture memory, language and executive attention). Contrary to the hypothesis of the study, acoustic stimulation failed to influence performance in all the cognitive domains investigated. Stimulation induced an increase in event-related potential (ERP) amplitude, while the intervals of stimulation were marked by a tendential sigma power increment, positively associated with memory consolidation. However, the experimental manipulation failed to affect spindle density and amplitude. Comparing the two experimental conditions, SWA resulted enhanced during blocks of stimulation and reduced during pause intervals, outlining a picture of unchanged overall NREM SWA. Additionally, in line with the study on healthy older adults [41], differences in SWA were obtained comparing periods of stimulation versus periods of pause during the stimulation night. Participants did not report an effect of the stimulation on their subjective perception of sleep quality, neither the sleep macrostructure was affected. Interestingly, despite the absence of cognitive improvements, the within-night SWA enhancement was associated both to over-night memory consolidation in WPAT and to the total score in the cognitive test battery. These results were consistent with the assumption that the reorganization of EEG power in the delta range could account for the acoustic stimulation effect on memory consolidation also in the aMCI condition. While this study failed to evidence the direct influence of sound stimulation on memory retention, the relation between cognitive performance and SWA increase during the stimulation periods supports the assumption of a preserved functional link between sleep slow waves and memory consolidation processes in the elderly population. Furthermore, the outcome encourages the future clinical application of the technique. 

Finally, a third replication study has been carried out in a group of middle-aged and older people [49]. In a within-subject design, seventeen healthy participants (mean age, 55.7; range 49–63) participated both in a night of closed-loop acoustic (50 ms) stimulation and in a control sham condition. Again, the pulse of sounds consisted of pink noise (50 ms), which were administered at an average of 54.5 dB, subjectively adjusted. Although the target of the stimulation was also in this case the up-state of the SOs, the authors adopted a different closed-loop system, which proved to be effective in young adults [37]. Starting from the detection of the down-state of the SO, the automated system presented a sound pulse after an individually determined delay (mean 583.24 ms) to hit the positive wave peak. Subsequently, a second sound was delivered at an individually determined interval (mean 1091.47 ms) in order to coincide with the positive peak of the second induced SO. Finally, a 2.5 second detection period followed the couple of sounds, during which the induced trains of SOs were observed. Another substantial difference with the above-mentioned similar works was the overall duration of the stimulation. Indeed, Schneider and colleagues [49] applied the sounds only for 3.5 hours from the first NREM stage detection, instead of the entire night [41,43]. As in Papalambros and colleagues [41,43], declarative memory was assessed with WPAT. Additionally, the authors tested the effects of acoustic stimulation on memory consolidation in the procedural domain and on post-sleep encoding abilities through a sequential finger tapping task (SFTT) and a picture encoding (PE) task, respectively. Unexpectedly, the experimental manipulation was associated with a worsening of overnight declarative memory consolidation compared to the sham condition, while SFTT and PE performance were unaffected. ERP analysis confirmed the ability of the first sound to elicit two additional SOs. Moreover, only the first induced up-state coincided with a fast spindle power enhancement. This pattern has been proposed to reflect stimulation-dependent spindle refractoriness, also found in the young population [37]. Spectral analysis revealed no effect of sound stimulation on SO and fast spindle bands. Experimental manipulation failed to increment the number and amplitude of SO in the stimulation period compared to sham. No effect on the architecture or perceived sleep quality was reported. Remarkably, Schneider and colleagues [49] compared their results with those previously obtained in a cohort of young adults [37], pointing to reduced responsiveness of the older group to this particular closed-loop approach. Elderly subjects were characterized by a substantially smaller SO amplitude and a lower stimulation-induced fast spindle power, as well as by a lower SO power and amplitude during the entire period of stimulation. Additionally, older subjects were characterized by stronger fast spindle refractoriness, which putatively reflects functional alterations within the thalamocortical system. In comparison to Papalambros and colleagues [41], the negative findings obtained could be in part attributable to the different closed-loop systems adopted. The PLL [40] seems to be more effective in the elderly population, due to greater temporal precision in the presentation of sounds. As was pointed out by Schneider and colleagues [49], another crucial difference between the two closed-loop systems was the default amplitude threshold according to which the sounds are administered. The PLL system is based on an average threshold of -40 μV, which allows to flexibly target oscillations not properly conceivable as SOs in line with the standard criterion [50]. Based on this evidence, PLL system optimizes the chance of stimulation in older people, characterized by a reduction of amplitude and duration of slow waves.

### 4.2. so-tDCS

The application of non-invasive electrical brain stimulation techniques during sleep is an effective approach to modulate cerebral physiology, successfully promoting SWA and sometimes spindle activity [36]. The tDCS consists in the supply of a low intensity electric current by means of two or more electrodes placed on the scalp. The current flow modulates the activity of the cortical area under the electrodes in a polarity-dependent manner, depolarizing the membrane potential of neuronal populations under the anodal electrode and hyperpolarizing those under the cathodal one. When the current intensity varies with a particular period, it is possible to modulate cortical activity in a frequency-specific manner. In a landmark study, Marshall and colleagues [51] showed that stimulation of frontal sites during early nocturnal NREM sleep through an anodal tDCS oscillating at the SO frequency (0.75 Hz) (so-tDCS) enhanced SO and slow sigma activity. Remarkably, these EEG effects during sleep were associated with the enhancement of declarative-memory consolidation, while non-declarative memories were not affected by the stimulation. Potential behavioral benefits of this approach were confirmed by several subsequent studies adopting similar methodologies in young healthy subjects and clinical populations [52,53] (for a meta-analysis, see [54]). Moreover, the beneficial effects of the protocol are frequency-specific. The same stimulation protocol applied with stimulation frequency in the theta range (5 Hz), another prevalent frequency band during early NREM sleep, results in opposite EEG and behavioral effects: a global reduction in SOs and a local decrease in slow sigma power, conjoint with the impairment of declarative memory consolidation [55]. According to the assumptions of the ASCH, SOs play a key role in sleep-dependent consolidation processes. Consequently, the effect of the so-tDCS is attributed to the capacity of the induced electrical potential fields to support action potential elicitation by prefrontal regions, typically involved in SO generation during sleep [29], promoting the entrainment of slow oscillatory activity. 

Based on the positive outcomes of the last years, the attention has recently shifted to the elderly population, which represents an attractive target due to the typical sleep alterations characterizing the aging process. To date, five studies [26,56,57,58,59] tested the efficacy of so-tDCS in older adults, assessing both the electrophysiological and cognitive domains (Table 1). Of note, the majority of studies have replicated the protocol of Marshall and colleagues [51], but the variations of timing or other parameters of the stimulation will be discussed in detail below. so-tDCS effects have been addressed adopting a counterbalanced randomized crossover design, whereby participants were exposed to stimulation and sham conditions. Usually, the stimulation begins during stage 2 of NREM sleep, and it is delivered in five 5-minute blocks, alternated by 1-minute intervals. The sites of the bifrontal stimulation are F3 and F4, cathodes are placed on mastoids, and the current is delivered at an intensity sinusoidally oscillating between 0 and 260–262.5 μA employing 8 mm circular electrodes, resulting in a maximum current density of 0.517–0.522 mA/cm^2^. 

Eggert and colleagues [56] were the first to extend the so-tDCS protocol to a population of twenty-six healthy elderly subjects (mean age, 69.1 years; range 60–90). Stimulation was carried out during early NREM sleep of an entire night. Of note, Eggert and colleagues [56] included in the stimulation protocol a current ramping period (8 seconds) at the beginning and end of the five stimulation blocks, in order to make the procedure less disruptive. Contrary to expectation, so-tDCS failed to improve declarative and procedural memory consolidation, addressed with WPAT and SFTT, respectively. Spectral analysis highlighted no stimulation-induced variation both during 1-minute stimulation-free intervals and during the entire night. Instead, the experimental manipulation led to a reduction in NREM stage 3, and an increment of awake time in the 1-minute interval, suggesting a putative disturbing effect of the practice. To account for these negative findings, it should be taken into consideration some consistent differences in the stimulation protocol compared to Marshall and colleagues [51] and the other four studies on elderly subjects. In Eggert and colleagues [56], 10 mm electrodes were used (vs. 8 mm of the other works), resulting in a lower current density of 0.331 mA/cm^2^. This may imply a putative insufficient current density to modulate the spontaneous cortical rhythms. 

Subsequently, Westerberg and colleagues [26] tested the efficacy of so-tDCS among nineteen older healthy subjects (mean age, 73.4 years; range 65–85) during a 90-minute nap. Again, the declarative domain was assessed with WPAT, accompanied by a fact recognition test. Additionally, an object-priming task was selected to measure non-declarative memory consolidation. A peculiarity of the Westerberg and colleagues [26] study was the sites of the current application, specifically F7 and F8, changing the current flow distribution in the brain. At the behavioral level, the stimulation-induced improvement was restricted to WPAT performance, confirming the ability of so-tDCS to promote the consolidation of more strictly hippocampal-dependent memories. This memory benefit was accompanied by a frontal SO activity enhancement and a reduction of fast spindle density over the central location in the 1-minute stimulation-free intervals compared to sham condition. No impact on SWS duration was reported, confirming the elusiveness of the classic sleep stage scoring to account for sleep-dependent memory consolidation processes in the elderly population. Moreover, no effect on the subjective quality of sleep was obtained. In line with Marshall and colleagues [51], the stimulation did not affect the rest of the sleep period. Although behavioral and EEG modifications were not directly related, this research proved the efficacy of so-tDCS during a nap in improving memory even in the elderly population, supporting a preserved functional link between sleep and declarative memory consolidation. 

Ladenbauer and colleagues [57] replicated the application of so-tDCS on the elderly population (18 subjects; mean age, 65 years) during a 90-minute nap, complementing the procedural (SFTT) and declarative (WPAT) memory assessment with a visuo-spatial task implying both visual and location memory. Differently to Marshall and colleagues [51], the 1-minute stimulation-free intervals were prolonged of further 40 seconds, which were not considered in the subsequent analyses, due to the long-lasting artifacts following stimulation. Moreover, both the duration of these intervals and the number of stimulation blocks were not fixed but adjusted according to the individual sleep characteristics. These peculiarities were introduced to ensure the stimulation during NREM stages 2, 3 or 4 (as in [53]), thus avoiding stimulating during undesired periods such as stage 1, REM sleep or wakefulness. Results showed a beneficial effect of the experimental manipulation on the picture recognition only, not on spatial memory, confining the impact of the practice to less complex aspects of declarative tasks [54]. Nevertheless, so-tDCS failed to improve WPAT performance. The negative finding was attributed to the greater semantic connection of the paired words compared to Westerberg and colleagues [26]. This feature configured a simpler task, which would have hindered the emergence of stimulation-induced effects since the hippocampal-dependent memory consolidation process is preferentially recruited in the reinforcement of weaker associations [60]. Again, no effect on sleep architecture was obtained neither within the stimulation-free intervals nor during the rest of the nap. At the electrophysiological level, so-tDCS led to an increment in SO and fast spindle frequency bands, as well as in fast spindle density. In light of these results, Ladenbauer and colleagues [57] confirmed the possibility to effectively modulate sleep physiology boosting declarative memory consolidation, although also in this case the sleep modifications were not directly correlated to memory consolidation improvement. 

Based on this evidence, the same group tested the efficacy of so-tDCS on an elderly population affected by aMCI [58]. Sixteen patients (mean age, 71.0 years; range 53–81) participated in an experimental protocol identical to Ladenbauer and colleagues [57] and were exposed to the same 90-minute nap stimulation procedure. The stimulation-induced memory benefit was limited to the picture recognition task also in the aMCI patients. This result has substantial clinical relevance because the recognition memory appears early impacted during the progression of AD [61,62], and the relative performance is a reliable predictive sign of healthy or pathological aging [63]. Spectral analysis showed that so-tDCS led to an enhancement in SO and spindle (slow and fast) frequency ranges power, confirming the results obtained in healthy older population [57]. Remarkably, a phase-amplitude coupling (PAC) analysis has been carried out by Ladenbauer and colleagues [58], showing a greater SO-fast spindle synchronization during stimulation-free intervals and an increased spindle power during the up-state of SOs compared to sham condition. On the other hand, sleep architecture was not affected by stimulation, except for an increase of stage 2 in the intervals without stimulation. In line with the previous literature [26,57], no correlation was obtained between changes in stage 2 and EEG power, and memory consolidation improvement. Noteworthily, declarative memory benefit was correlated with the enhanced synchronized coupling between SO and fast spindle, which occurred during the stimulation condition. This finding extends to aMCI patients the pivotal functional role of the synchronization between different brain rhythms (cortical SOs and thalamocortical spindles) to support sleep-dependent memory consolidation. 

Finally, Paßmann and colleagues [59] tested the efficacy of so-tDCS on twenty-one healthy older subjects (mean age, 65 years) during the first NREM period of an entire night (as in [56]), adopting the stimulation procedure of Ladenbauer and colleagues [57]. In line with precedent literature [26,57,58], stimulation led to a SO power increment in the stimulation-free intervals, and this effect was found also one hour after the so-tDCS application. Moreover, the experimental manipulation induced an increase of EEG activity in the range of both slow and fast spindles compared to the sham condition. Surprisingly, the modulation of these sleep parameters was not followed by memory consolidation benefits. Instead, overnight visual memory performance was even negatively affected. Moreover, sleep scoring analysis showed a tendential increment of wake time within the intervals between the stimulation blocks, and the rest of the night was marked by less stage 4 NREM following so-tDCS. In line with Eggert and colleagues [56], these outcomes confirm a putatively short-term fragmentation effect of the stimulation, which could affect the subsequent overnight deep sleep phases, explaining the impaired memory consolidation.

## 5. Conclusions

The current literature suggests minimal invasiveness of the sound stimulation approach, capable of modulating sleep electrophysiology in the elderly population without impacting sleep architecture as well as the subjective quality of sleep. Furthermore, the three available studies confirmed feasibility and effectiveness to use closed-loop auditory stimulation systems in older people, notwithstanding the typical morphological slow wave alterations, which characterize both healthy and pathological aging. In this regard, it should be noted that the current literature simply applied to the elderly the same protocols successfully adopted in young adults. Nevertheless, the optimal stimulation parameters differed between young and older subjects [64]. Further investigations on the elderly population are warranted to determine ad hoc stimulation protocols, with a special focus on the appropriate stimulation timing. Accordingly, the stimulation delivered closer to the SO peak has proved to be more powerful to induce SO enhancement [64]. Consistently, Papalambros and colleagues [41] showed that more consistent benefits on declarative memory were obtained by participants who received the sound stimulation in the phase closer to the SO up-state (mean, 7.9 degrees vs. 17.7 degrees before the SO positive peak). Additionally, the best targeting amplitude threshold of SOs remains a subject for future investigations, considering that it could be a crucial parameter in determining the final outcome of the stimulation. A final consideration concerns the stimulation intensity. While Papalambros and colleagues [41,43] adjusted the sound pressure level according to the subjective rating of the participants during the awake state, Schneider and colleagues [49] determined the sound intensity during a precedent adaptation night. Although in the latter study no memory benefit has been reported, the individuation of procedures to systematically adjust the sound intensity according to the subjective acoustic threshold should be necessary to define more reliable and generalizable stimulation protocols. Moreover, in the current literature a fixed acoustic intensity during the entire stimulation period has been adopted, although the tuning of the sound volume according to the sleep depth could be a practical approach to optimizing the stimulation outcome [32].

On the other hand, the literature on the so-tDCS application in older people is wider and more variegated. so-tDCS appears to be a safe, non-invasive, and well-tolerated approach. Except for Eggert and colleagues [56], the current literature coherently assigned to the practice the potential to manipulate sleep electrophysiology, successfully boosting SO power. The inconsistent results by Eggert and colleagues [56] allow us to shed light on a crucial methodological issue, such as the choice of the current density. The authors [56] significantly reduced the current density of the stimulation compared to the rest of literature both on older [26,57,58,59] and young people [51]. This feature could have undermined the effectiveness of the practice, invalidating the capacity of the stimulation to synchronize the cerebral activity. Furthermore, a peculiarity of Eggert and colleagues [56] was the employment of a current ramping procedure at the beginning and end of the stimulation periods. Nevertheless, it is difficult to imagine how this protocol supplementation could have negatively impacted the stimulation outcome since the stimulation duration was even increased.

Another crucial point is the ongoing assessment of the sleep stage of stimulation. Adopting a state-dependent stimulation approach could be a profitable strategy to optimize the effectiveness of the practice, considering the growing evidence of the brain state dependency of the stimulation efficacy [55,65]. This could be particularly prominent in studies on older people, which are marked by consistent sleep fragmentation. Actually, the stimulation protocol used in Ladenbauer and colleagues [57,58] and Paßmann and colleagues [59] successfully led to both an increment of SO and spindle activity, and in one case also of the density of the spindle events [57]. Of note, notwithstanding its potential advantages, this stimulation approach is incompatible with a double-blind protocol, since the experimenters can hardly be kept in the dark about the real ongoing experimental condition (stimulation or sham). 

Other substantial inconsistencies in the current literature seem to be connected to the application of the so-tDCS during a nap or an early NREM period of the night. The memory benefits were reported only in nap studies [26,57,58]. On the other hand, the two overnight studies [56,59] reported an immediate disturbing effect of the stimulation as well as a negative impact on subsequent deep sleep stages. These adverse consequences may compromise, and even reverse, the short-term cognitive benefits induced by the stimulation, supporting the speculative hypothesis of naps as optimal periods to avoid overnight side effects, leading to memory improvements in the elderly population [59]. Future investigations should fine-tune the so-tDCS overnight application in order to limit the negative consequences on sleep architecture. In this view, it has been proposed [59] to reschedule the stimulation interval from the first to the subsequent NREM cycles, which imply more stable and longer SWS in older people [66]. 

Finally, the exact electrophysiological correlates of the reported cognitive benefits induced by so-tDCS are still unclear. For example, some works reported a positive effect of the stimulation on both slow and fast spindles [58,59], while others a negative influence [26]. The mixed findings suggest the importance of confirming the results within larger samples. In particular, future research should pay more attention to the temporal coupling between SO and spindle events since it could have a pivotal role to account for the memory effects, as shown in Ladenbauer and colleagues [58]. Nevertheless, recent evidence suggested a possible impairment of this functional relationship in older subjects [31]. In this view, further investigations will allow us to deepen our understanding of the complex dynamics underlying sleep-dependent memory consolidation during aging.

A general consideration involving both acoustic and electrical stimulation concerns the tasks used to assess cognitive functions. The literature is characterized by inconsistencies on the behavioral side, commonly ascribed to the use of different versions of the same task (e.g., WPAT). The only way to rule out this confounding factor should be employing fully identical and comparable tasks, so as to shift the focus on the specific effects of the different stimulation protocols. 

In conclusion, the exogenous promotion of SOs during sleep aimed at improving declarative memory function in the elderly population could be a promising research field for potential everyday life and therapeutic applications. Nevertheless, the current literature is limited and presents inconsistencies in the cognitive and electrophysiological outcomes, which appear to be extremely sensitive to the protocol adopted, limiting the generalization of the effects. Different results were obtained according to the stimulation parameters, the task engaged, and the stimulation periods. This evidence highlights the importance of confirming the current results on large samples, identifying the most effective protocol in order to allow the older people to benefit from these neurostimulation procedures. Moreover, future research should explore the electrophysiological and cognitive long-term effects of these stimulation approaches as well as the consequences of prolonged application periods to test the hypothesis of a cumulative benefit of the two stimulation techniques, evaluating potential side effects in healthy and clinical populations. 

Remarkably, both kinds of stimulation are proven to be effective and feasible in aMCI patients [43,58]. This evidence paves the way for the application of these practices in pathological aging. For this purpose, randomized controlled trials are needed to transfer these procedures to clinical settings. SO enhancement during sleep may be potentially applied in the treatment and prevention of cognitive symptoms of neurodegenerative conditions. Furthermore, slow oscillatory activity was proven to be implicated in the clearance of β-amyloid protein during sleep [67], whose accumulation is the basis of the pathogenesis of AD [68]. On the other hand, SWA disruption is closely connected to the aforementioned toxic peptide aggregation [69,70], exacerbating the onset of the disorder. In light of the growing evidence supporting the assumption of a bidirectional relationship between AD and sleep disruption [69,71], a more ambitious and fascinating implication may be the potential therapeutic application of these techniques to directly counteract the progression of the physiological symptomatology characterizing some clinical conditions linked to pathological aging. However, this speculation should be considered with caution since the neurostimulation procedures described in this review could be effective in manipulating only the mere manifestation (i.e., the slow wave decline) of the underlying neurodegeneration.

## 6. Future Directions

Whereas both slow-oscillatory acoustic and electric stimulations appear to be effective approaches in the older population, it is more difficult to imagine for so-tDCS a displacement from the laboratory setting due to its intrinsic methodological limitations (e.g., setup complexity and potential side effect for a long-lasting application) [36]. On the other hand, all the studies adopting an acoustic stimulation approach in older people focused their SO detection algorithms on a single EEG channel (i.e., Fpz). This feature offers a concrete perspective to translate this approach to the home setting using simplified wearable EEG devices, allowing to test the efficacy of the practice for protracted periods in a purely ecological setting. In line with this idea, Debellemaniere and colleagues [72] successfully applied to young adults a portable EEG device implemented with an automated closed-loop system to deliver pulses of pink noise during the transition from the down- to the up-state of SOs. These stimulations led to an increment in SO amplitude, which was also confirmed after ten days of home stimulations. Similarly, the effectiveness of another portable EEG system was confirmed in young adults [73], showing that the at-home enhancement effect was extended to spindle power. More recently, another study [74] adopting a wearable device (SmartSleep, Philips Healthcare) in middle-aged man (mean age, 39.9 years; range 35–48) showed a significant stimulation-induced SWA enhancement, which was correlated to some cognitive benefits (verbal fluency and working memory). Although we are only at the beginning, these findings support the perspective of a near future where the elderly population could benefit from non-invasive acoustic stimulation at home for extended periods, putatively counteracting the cognitive and sleep quality declines which characterize both healthy and pathological aging. Further investigations are warranted to confirm the safety profile of the prolonged application of these stimulation techniques, evaluating potential side effects. 

In the last years, other sensorial stimulation approaches have been used to modulate the physiology of the sleeping brain and potentially improve cognition (for review, see [1,36]). Vestibular stimulation (i.e., slow bed rocking (0.25 Hz)) has been successfully applied to boost SOs and spindle activity [75,76], linked to a benefit on declarative memory consolidation [76]. Moreover, odor delivery (e.g., lavender oil scent) has proved to induce SWA and spindle enhancement [77]. Vestibular and olfactory stimulations during sleep may prove to be a winning approach in older people, allowing to circumvent the intrinsic limit of hearing impairments that are frequently encountered within this population, and that complicates the possibility of benefiting from acoustic stimulation. Additionally, odor stimuli processing bypasses thalamic structures, which are typically involved in arousal generation [78]. This evidence suggests a putative lower likelihood of the olfactive stimulation to induce awakenings and disrupt sleep architecture [79]. Finally, since odor delivery does not imply sleep interruption and K complex responses at variance with other sensory modality [77], it could be a more punctual approach to examine the physiological effects induced by sensory stimulations during sleep. Despite the practical and theoretical advantages, the exact underlying functional mechanisms are not clear, and the evidence on the cognitive side is extremely limited. Future studies could address these open questions in order to proceed with the application of such stimulation protocols to older people. 

Other transcranial stimulation techniques have been successfully applied during sleep to boost the SOs in young subjects. Massimini and colleagues [80] showed that the cortical perturbation during NREM sleep through rTMS (with pulse frequency of ~0.8 Hz) triggered SOs, also influencing the spindle density. Magnetic stimulation may not be appropriate for prolonged use among the older population, considering that it may be associated with more physical discomfort and arousal compared to electric stimulation procedures. Nevertheless, it may prove particularly useful in experimental settings, considering the higher spatial resolution. Additionally, the EEG could be recorded concurrently with the pulses of stimulation. These features outline a greater spatial and temporal accuracy in the identification of the induced effects. 

Furthermore, recent studies reported the efficacy of closed-loop tACS protocols to promote SOs during sleep in young adults [81,82]. These procedures positively influenced overnight visual memory consolidation, showing for the first time the efficacy of tACS to modulate the slow waves of the sleeping brain matching the stimulation frequency and phase with ongoing slow-wave activity. tACS could be a promising technique in order to induce the entrainment of sleeping brain oscillations since it allows to directly manage both depolarizing and hyperpolarizing phases of the slow waves [83]. Furthermore, these works [81,82] provide the first evidence of closed-loop system employment to optimize transcranial electric stimulation. This approach could be highly functional to optimize stimulation protocols in older people, considering the brain state dependency of the stimulation efficacy. Consistently, Cellini and colleagues [84] introduced a hybrid approach, adapting the classic so-tDCS protocol [51] to a closed-loop system, which was adopted previously in a sound stimulation paradigm [85]. They delivered a short-duration electric stimulation during NREM stages 2 and 3 according to the SO detection, which resulted in a persistent performance improvement in a declarative memory task accompanied by an overnight increment of SO rate and SWS duration. Of note, a feedback-controlled system within an electric stimulation protocol had already been implemented by Lustenberger and colleagues [86], though for a different scope. In this case, brief current pulses with a characteristic spindle-like waveform (12 Hz) were delivered matching the detection of spindle events. This procedure led to a general increment in the spindle activity during N2, which was correlated to the significant positive impact on procedural memory consolidation (SFTT). Lustenberger and colleagues [86] introduced the possibility to directly promote spindle activity in the sleeping brain through an ad hoc protocol of stimulation. Remarkably, the same goal has been reached presenting acoustic stimuli at peculiar frequencies (12 and 15 Hz) during NREM sleep [87], even though the results were not confirmed by a recent study which adopted a closed-loop system [88]. In light of the current literature, future investigations could test innovative and more targeted stimulation protocols aimed to directly boost both SO and spindle activity, as well as their functional coupling. This could turn into a further advantage on the cognitive side, with important implications particularly for the elderly population.

## Figures and Tables

**Table 1:** List of current studies applying acoustic or electric stimulation protocols to boost slow oscillations and improve sleep-dependent memory consolidation in elderly population.

Study	Population	Stimulation Type	Stimulation Time Window	Stimulation Parameters	Electrophysiological Outcomes	Cognitive Outcomes
Papalambros et al., 2017 [41]	13 healthy subjects (mean 75.2 years; range 60–84)	PLL acoustic stimulation	Overnight	**Stimulus**: 50 ms pink noise (30–50 dB) **Procedure**: Blocks of five pulses (ISI mean 1.2 s), alternated with stimulation-free blocks (∼6 s) **Period**: NREM sleep of the entire night	↑ ERP amplitude increase ↑ SWA (stimulation vs. stimulation-free blocks) ↑ spindle density and amplitude	↑ WPAT WPAT memory improvement positively correlated with SWA increment in stimulation vs. stimulation-free blocks
Papalambros et al., 2019 [43]	9 aMCI subjects (mean 72 years; range 62–86)	PLL acoustic stimulation	Overnight	**Stimulus**: 50 ms pink noise (30–50 dB) **Procedure**: Blocks of five pulses (ISI ∼1 s), alternated with stimulation-free blocks (∼6 s) **Period**: NREM sleep of the entire night	↑ ERP amplitude increase ↑ SO activity (stimulation vs. stimulation-free blocks) ↑ SWA (stimulation vs. stimulation-free blocks)	↔ WPAT ↔ NIHTB Overnight performance change in WPAT positively correlated with SWA and SO activity increment in stimulation vs. stimulation-free blocks
Schneider et al., 2019 [49]	17 healthy subjects (mean 55.7 years; range 49–63)	CL acoustic stimulation	Overnight	**Stimulus**: 50 ms pink noise (mean 54.5 dB) **Procedure**: Two pulses (ISI mean 1091 ms), followed by 2.5 s without stimulation **Period**: 3.5 hours from the first stable NREM sleep	↑ ERP amplitude increase ↔ SO activity ↑ fast spindle activity phase-locked to induced SO up-states	↓ WPAT ↔ SFTT ↔ Picture encoding
Eggert et al., 2013 [56]	26 healthy subjects (mean 69.1 years; range 60–90)	so-tDCS	Overnight	**Stimulus**: Anodal current sinusoidally oscillating at 0.75 Hz between zero and 260 μA (current density, 0.331 mA/cm^2^), delivered on F3 and F4 **Procedure**: 5 five-min stimulation blocks, alternated by fixed 1-min stimulation-free blocks (8 s current ramping period at the beginning/end of the stimulation blocks) **Period**: From the first NREM sleep	↔ SO activity ↑ Wake in stimulation-free blocks ↓ NREM stage 3 in stimulation-free blocks	↔ WPAT ↔ SFTT
Westerberg et al., 2015 [26]	19 healthy subjects (mean 73.4 years; range 65–85)	so-tDCS	Nap	**Stimulus**: Anodal current sinusoidally oscillating at 0.75 Hz between zero and 260 μA (current density, 0.517 mA/cm^2^), delivered on F7 and F8 **Procedure**: 5 five-min stimulation blocks, alternated by fixed 1-min stimulation-free blocks **Period**: From the first stable NREM sleep stage 2	↑ SO activity ↓ central fast spindle density	↑ WPAT ↔ Fact recognition task ↔ Object-priming task
Ladenbauer et al., 2016 [57]	18 healthy subjects (mean 65 years)	so-tDCS	Nap	**Stimulus**: Anodal current sinusoidally oscillating at 0.75 Hz between zero and 260 μA (current density, 0.522 mA/cm^2^), delivered on F3 and F4 **Procedure**: 3–5 five-min stimulation blocks, alternated by (at least) 100 s stimulation-free blocks **Period**: From the first stable NREM sleep stage 2, ensuring to start the subsequent stimulation blocks only during stage 2–4	↑ SO activity ↑ fast spindle activity and density	↔ WPAT ↔ SFTT ↔ Visuo-spatial task (location memory) ↑ Visuo-spatial task (visual memory)
Ladenbauer et al., 2017 [58]	16 aMCI subjects (mean 71 years; range 53–81)	so-tDCS	Nap	**Stimulus**: Anodal current sinusoidally oscillating at 0.75 Hz between zero and 262.5 μA (current density, 0.522 mA/cm^2^), delivered on F3 and F4 **Procedure**: 3–5 five-min stimulation blocks, alternated by (at least) 100 s stimulation-free blocks **Period**: From the first stable NREM sleep stage 2, ensuring to start the subsequent stimulation blocks only during stage 2–4	↑ SO activity ↑ fast and slow spindle activity ↑ SO-fast spindle activity phase amplitude coupling ↑ spindle activity phase-locked to SO up-states ↑ NREM stage 2 in stimulation-free blocks	↔ WPAT ↔ SFTT ↔ Visuo-spatial task (location memory) ↑ Visuo-spatial task (visual memory) Overnight visual memory improvement positively correlated with increment in the SO-fast spindle activity coupling
Paßmann et al., 2016 [59]	21 healthy subjects (mean 65 years)	so-tDCS	Overnight	**Stimulus**: Anodal current sinusoidally oscillating at 0.75 Hz between zero and 260 μA (current density, 0.522 mA/cm^2^), delivered on F3 and F4 **Procedure**: 5 five-min stimulation blocks, alternated by (at least) 100 s stimulation-free blocks **Period**: From the first stable NREM sleep stage 2, ensuring to start the subsequent stimulation blocks only during stage 2–4	↑ SO activity ↑ fast and slow spindle activity ↓ NREM stage 4	↔ WPAT ↔ SFTT ↔ Visuo-spatial task (location memory) ↓ Visuo-spatial task (visual memory)

**Abbreviations**: aMCI = amnestic mild cognitive impairment, PLL = phase-locked loop, CL = closed-loop, so-tDCS = slow oscillatory-transcranial direct current stimulation, ISI = inter-stimulus interval, WPAT = word-paired association task, SFTT = sequential finger tapping task, ERP = event-related potential, SO = slow oscillation, SWA = slow wave activity, NREM = non rapid eye movement sleep, NIHTB = National Institutes of Health Toolbox Cognition Battery. ↑ = increase; ↔ = unchanged; ↓ = decrease.
